# Mapping hypersensitivity/allergic diseases in the International Classification of Diseases (ICD)-11: cross-linking terms and unmet needs

**DOI:** 10.1186/s13601-015-0063-x

**Published:** 2015-06-03

**Authors:** Luciana Kase Tanno, Moises Calderon, Nikolaos G. Papadopoulos, Pascal Demoly, on behalf of the EAACI/WAO Task force of a Global Classification of Hypersensitivity/Allergic diseases

**Affiliations:** Hospital Sírio-Libanês, granted by CNPq, São Paulo, Brazil; Section of Allergy and Clinical Immunology, Imperial College London, National Heart and Lung Institute, Royal Brompton Hospital, London, UK; Centre for Paediatrics and Child Health, Institute of Human Development, University of Manchester, Manchester, UK; Department of Allergy, 2nd Pediatric Clinic, University of Athens, Athens, Greece; Department of Pulmonology - Division of Allergy, University Hospital of Montpellier, Montpellier, and Sorbonne Universités, UPMC Paris 06, UMR-S 1136, IPLESP, Equipe EPAR, 75013 Paris, France

**Keywords:** Allergic Diseases, Hypersensitivity, Classification, International Classification of Diseases (ICD), Cross-Linking

## Abstract

**Background:**

With the aim of actively contributing to the ongoing 11^th^ International Classification of Diseases (ICD) revision, an international collaboration led by the European Academy of Allergy and Clinical Immunology (EAACI) has decided to revise the classification of hypersensitivity/allergic diseases and to validate it for ICD-11 by crowdsourcing the allergist community. However, understanding that the construction of a classification was necessary but not sufficient, we developed a mapping strategy in the attempt to better fit it to the ICD-11 linearization structure.

**Methods:**

The cross-linking terms process has been constructed based on an algorithm in which we prioritized the pre-coordination, followed by the post-coordination when the first step was not possible. If the above strategies failed to identify the entries, the conditions were ruled as “non specific terms”, “no code fit properly” or “missing terms”.

**Results:**

Amongst the 652 terms distributed in 5 main groups of the Hypersensitivity/Allergic Diseases classification, 169 terms fit directly the codes listed in the ICD-11 beta draft (October 2014 version), 26 were considered as “nonspecific term”, 21 were linked to the Foundation by Index, 7 were recorded as inclusions and 2 were cited just in the definition of the condition. The post-coordination was possible for 97 terms, mainly for drug hypersensitivity conditions. We noticed a considerable number of allergen references missing.

**Conclusion:**

The proposed strategy of cross-linking terms and the results of this process can actively contribute to updating the hypersensitivity and allergic conditions classification in the ICD-11 beta revision and underlines the need for either a new chapter in ICD-11 possibly entitled Hypersensitivity / Allergic Disorders or at the very least the aggregation of all such diseases under the “Diseases of Immune System” chapter in order for the overlaps to be double parented to the appropriate ‘system’ chapters.

## Background

### The need of actively supporting the ICD-11 revision for a better hypersensitivity/allergic diseases coding

#### The classification of diseases for a better coding

Assessment and models of health have been constructed around the medical classification “language” to better understand health-disease balance and outcomes as well as to support evidence-based decision-making. It has created the need for mechanisms and tools to capture, analyze, interpret and monitor medical data. This need has given rise to a process of translating the names of clinical disorders into codes to assist the collection, later analysis and comparability. However, some recognized cornerstones to achieve a reliable coding system organization are to have broadly accepted terminologies and a classification of clinical disorders able to reflect the clinical practice and globally endorsed and used by specialists and non-specialists. Many different coding systems have emerged, such as the Diagnostic and Statistical Manual of Mental Disorders (DSM) for the eponym diseases, and more broadly the Systematized Nomenclature of Medicine-Clinical Terms (SNOMED-CT) and the International Classification of Diseases (ICD). The coding systems have been constructed under different structures with specific terminologies to facilitate the applicability by distinctive groups of users [1–3].

The ICD, maintained by the World Health Organization (WHO), proposes to provide a common language for reporting and monitoring diseases to achieve the standard of being a universal classification [1]. However, we have demonstrated the inadequacy of the ICD-10 and the current ICD-11 beta phase structures (May and October 2014 versions) to represent the hypersensitivity/allergic diseases classification, as reported by the allergist community [4]. As the most striking example, the ICD-10 showed to be unable to recognize anaphylaxis deaths as the underlying cause of deaths [5] and after a careful comparison between ICD-10 and 11 beta phase linearization codes we identified gaps and trade-offs allowing us to bring up the need of a higher structure for these conditions [3].

#### The ICD-11 beta draft structure and updates

With the ICD revision, WHO seeks a scientific basis to ensure comparability and consistency and to allow flexibility of this tool to be fit for different purposes. The ICD-11 beta draft is an online platform launched in 14^th^ May 2012, which receives constant inputs and is updated regularly. A pre-final version will be tested in real life in 2015. The final version of the ICD-11 is expected to be presented to the WHO’s World Heath Assembly in 2017. In the mean time, the beta phase content is continuously reviewed by scientific peers and updated accordingly what permits additions, deletions and changes [6].

The ICD-11 platform has been created based on a digital library including all ICD entities called *Foundation Component*, which is the basis of the construction of a user-defined list, called *Linearization*. The Foundation Component is composed by all attributes of a health condition outlined by a content model and the Linearization is, therefore, a subset of the Foundation Component built to fit a particular purpose such as primary care, reporting morbidity, mortality among other uses. The Linearization is composed by entities that are mutually exclusive, meaning that each entity receives a single parent and reflects the final structure of the forthcoming ICD-11 structure. The ICD conditions listed in the Foundation can be linked to the online beta draft linearization by Index citation. The current linearization (October 2014 version) counts with 26 chapters [1, 6].

Some of the ICD-11 beta draft updates regarding the Allergy and Clinical Immunology field included the creation of a “Diseases of the Immune System” chapter (first noticed in May, 2014) and a code for Allergy and allergic reactions (ND51.2 last viewed in the ICD-11 linearization November version) under the “External Causes” chapter. After sharing the classification of hypersensitivity/allergic diseases with representatives of Dermatology, Rare Diseases, Pediatric, Internal Medicine and Ophthalmology Topic Advisory Groups (TAGs) (July 2014), more improvements in relation to Allergy have been noticed, such as (1) a formal proposal to add a category for conditions due to adverse immune response into the “Diseases of the Immune System” chapter from multiple parented primary locations, if relevant and (2) to include categories such as ‘food allergy’ or ‘adverse food reaction (September 2014).

#### Terminologies for hypersensitivity/allergic diseases

“Terms” are words or group of words responsible for providing a specific meaning according to the context they are used, being able to be considered stable identifiers. “Nomenclature” is a system of terms, constructed according to a specific rule, in a particular field of science, making possible labeling and designating *concepts*. When a term is created, a set of words is coordinated together into a concept to represent some piece of relevant information.

Based on the similarities and differences of the conditions, which receives terms, it is possible to delineate a classification. However, the terms have to be adapted according to the end-users of the classification. In this way, the EAACI-WAO survey of health professionals’ attitudes toward allergic disorders classification [4] demonstrated the need of a global cross-culturally applicable classification system of allergic diseases.

In the Allergy specialty, the constant generation of new knowledge forces the update of concepts and, therefore, nomenclature/terms; in this regard the above survey showed that the EAACI-WAO revised nomenclature [7] is accepted by the majority of the allergist community. Based on that, *“hypersensitivity”* is defined as “conditions clinically resembling allergy that cause objectively reproducible symptoms or signs, initiated by exposure to a defined stimulus at a dose tolerated by normal subjects” and “*allergy”* as “a hypersensitivity reaction initiated by immunologic mechanisms”. However, this distinction is still not used by non-allergists and non-physicians; consequently, the solution we used was always to link the terms *hypersensitivity* and *allergy*. Although we are aware of the importance of immunological diseases including immune deficiencies and autoimmune disorders, these conditions were not incorporated into the current document since the ICD-11 has already dedicated a chapter for these disorders entitled “Immune System Disorders” [6].

#### The new classification of hypersensitivity/allergic diseases

**Fig. 1:**
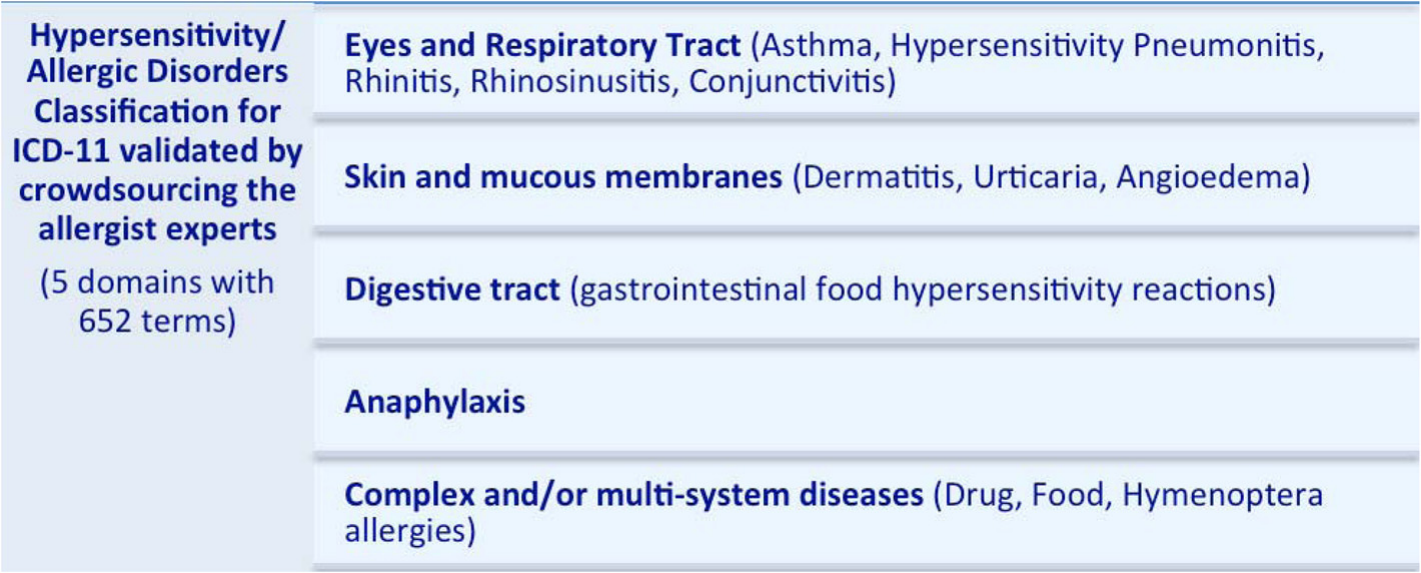
General structure of the Hypersensitivity/Allergic Classification for ICD-11 validated by crowdsourcing the allergist experts

With the aim of actively contributing to the ongoing 11^th^ ICD revision, an international collaboration led by the European Academy of Allergy and Clinical Immunology (EAACI) has decided to revise the classification of hypersensitivity/allergic diseases and to validate it for ICD-11 by crowdsourcing the leaderships of the major allergy societies across the world, experts in allergy, terminology leaders and end-users [8]. As a result, a high level complex structure of classification for hypersensitivity/allergic diseases has been constructed with 10 main categories of hypersensitivity/allergic diseases covering respiratory (asthma and rhinitis), skin (dermatitis, urticaria and angioedema), ocular (conjunctivitis), drug, food and Hymenoptera venom allergies and anaphylaxis distributed in 5 main groups (Fig. 1). The document has been shared with a SNOMED CT terminology expert, Topic Advisory Group (TAGs) and the Revision Steering Group (RSG) representatives in charge of the ICD revision (July 2014), with whom we have been carrying out from the beginning bilateral discussion to have the endorsement of the classification proposal for the ICD-11.

Advised by representatives of some TAGs and the RSG and believing that more than offering the classification proposal, we could contribute to aligning the hypersensitivity/allergic diseases classification to the ICD-11 beta draft linearization facilitating the classification proposal acceptance, we here propose the process of mapping the constructed classification of hypersensitivity/allergic in the current ICD-11 beta draft linearization (October 2014 version) by cross-linking terms and highlighting the unmet needs.

## Methods

### Interactive mapping of hypersensitivity/allergic diseases in the ICD-11

#### Methods to build the cross-linking terms

**Fig. 2:**
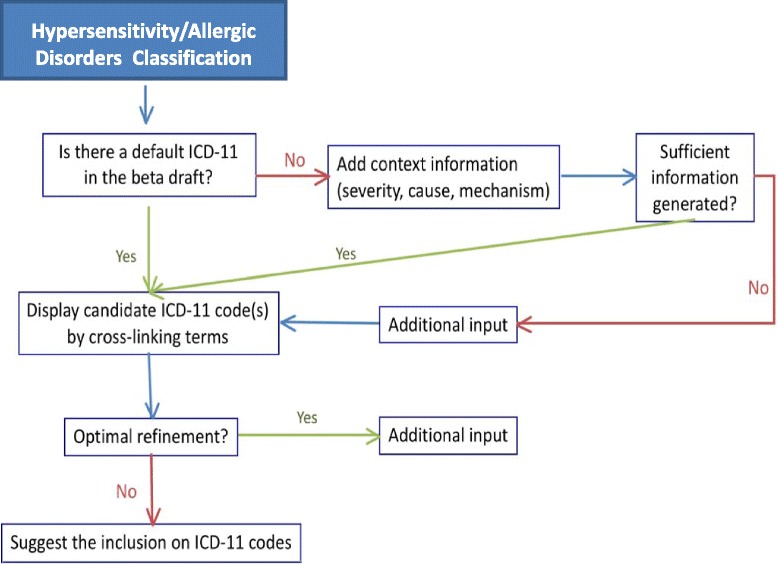
Algorithm of Interactive Mapping Assistance Generation of Allergic Diseases ICD Codes strategy

**Fig. 3:**
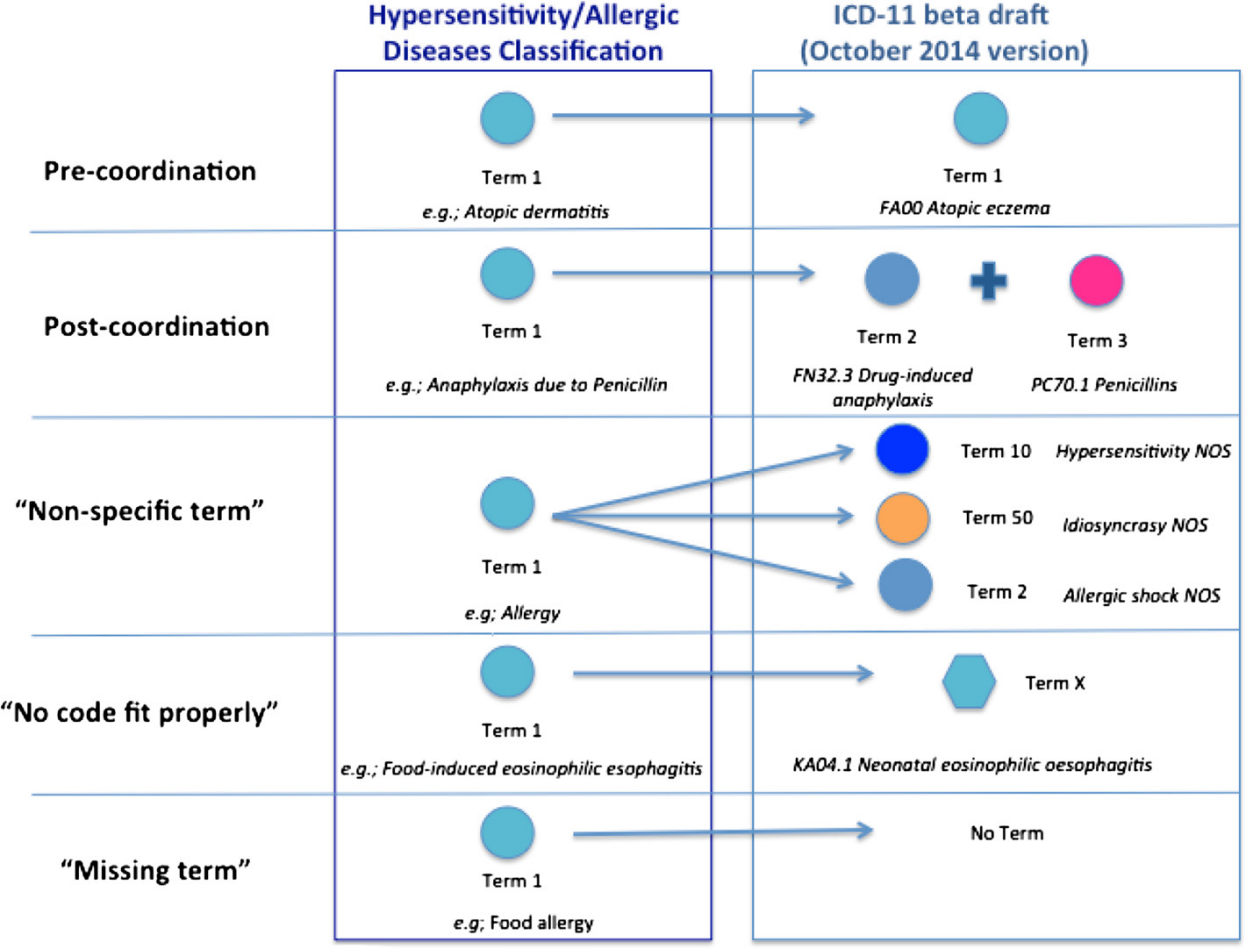
General scheme of the classification strategy

Understanding that the construction of a classification of hypersensitivity/allergic diseases by crowdsourcing the allergy community was necessary but not sufficient, we have addressed the question of how to fit the hypersensitivity/allergic diseases entries to the current ICD-11 framework. To answer this query, we developed a search strategy (Figs. 2 and 3). For that, the published classification proposal [8] and the online ICD-11 beta draft linearization framework (October 2014 version) [6] were considered as the basis of the searching process. We carefully looked at all the terms of the validated hypersensitivity/allergic diseases classification, checked if they were cited in the ICD-11 beta draft and classified the terms as able to “pre-coordination”, “post-coordination”, “non-specified term”, “no code fit properly” and “missing term”.

In the first step of the process, believing that all hypersensitivity/allergic diseases deserve their own codes, the pre-coordination was prioritized. In this way, all possible corresponding codes for each hypersensitivity/allergic condition described in the classification proposal were listed according to the algorithm shown on Fig. 1. “Pre-coordination” is the fixed elements into one heading in anticipation of a search on that compound heading. It is needed to avoid disambiguation and/or suggestibility besides facilitating an accurate search in a health classification system. The pre-coordination is preferable once the most useful mappings are the one-to-one maps, in which a single hypersensitivity/allergic concept can be used to represent the full meaning of an ICD-11 beta draft code/term. This allows the automatic translation of Hypersensitivity/allergic conditions into ICD-11 codes without loss of meaning.

In cases in which pre-coordination was not possible, post-coordination was considered. “Post-coordination” is the combining or adding to an existing entity or keyword additional details to provide greater specificity to the entity. These additional details can provide more information such as severity, causality, mechanism and anatomy. The basis of the combining process usually obeys the recommendations of the WHO and the Library of Congress Subject Headings [1, 6, 9]. In this system, many headings can be constructed from individual elements that represent clinical presentation, cause, site or underlining mechanism (*e.g.,* “drug induced-anaphylaxis” as the condition + “Penicillin” as the cause). In practice, it can be used for complex and multi-element conditions to achieve coverage; however, without some rational grammar an end-user can post-coordinate an invalid notion from the available parts.

If the above strategies failed to identify the hypersensitivity/allergic entries, the conditions were labeled as “non specific terms” when the existent term was too generic or able to cover many different conditions, such as the “catch-all” codes that encompass heterogeneous diseases or conditions (*e.g.*, “other specified papillary conjunctivitis”). We considered as “missing terms” cases in which the corresponding terms were completely absent, and “no code fit properly” when the ICD-11 classification did not cover exactly the hypersensitivity/allergic condition (*e.g.,* KA04.1 Neonatal eosinophilic oesophagitis” for Food-induced eosinophilic esophagitis).

All the process of searching the key terms correspondences into the ICD 11-beta draft platform (October 2014 version) had an online basis. It was, then validated by LKT and PD and cross-checked to avoid disambiguation and dubious interpretation of data. We are, however, aware that the definitive codification is not established in the linearization up to now.

## Results and discussion

### Results of the cross-linking terms process

Considering the main definitions of “hypersensitivity” and “allergy”, we first looked for these terms in the online ICD-11 beta draft. As a result, we observed that both terms are currently listed 9 times each across the ICD-11 structure (Table 1).

**Table 1:** Mapping the terms “hypersensitivity” and “allergy” in the ICD-11 beta draft structure (October 2014 version)

Terms	Definition according to the EAACI-WAO revised nomenclature	ICD-11 Beta draft search (October 2014 version)	Location according to the Linearization (October 2014 version)
“Hypersensitivity”	“Conditions clinically resembling allergy that cause objectively reproducible symptoms or signs, initiated by exposure to a defined stimulus at a dose tolerated by normal subjects.”	“Hypersensitivity reactions”	Under the “Disorders of lymphocyte number” subchapter
		“Hypersensitivity myocarditis”	Under the “Diseases of the myocardium” chapter
		“Hypersensitivity pneumonitis due to organic dust”	Under the “Pneumonia” subchapter
		“Other specific hypersensitivity pneumonitis due to organic dust”	
		“Hypersensitivity pneumonitis due to organic dust, unspecified”	
		“Hypersensitivity of the labyrinth”	Listed as an Inclusion of “Labyrinthine dysfunction”
		“Drug-induced hypersensitivity syndrome”	Listed as an Inclusion of “DRESS Syndrome”
		“Hypersensitivity NOS”	Listed as an Inclusion of “Adverse effects, not elsewhere classified”
		“Skin tests for: hypersensitivity”	Cited as an Inclusion of “Diagnostic skin and sensitization tests”
“Allergy”	“A hypersensitivity reaction initiated by immunologic mechanisms.”	“Allergy to substances in contact with the skin”	Under the “Skin disorders provoked by external factors” subchapter
		“Oral allergy syndrome”	Under the “Allergy to substances in contact with the skin” subchapter
		“Other specified forms of cutaneous allergy”	
		“Allergy or allergic reaction”	Under the “Adverse effects, not elsewhere classified” subchapter
		“Allergy NOS due to pollen”	Under the “Vasomotor and allergic rhinitis” subchapter
		“Pollen-food allergy syndrome”	Cited as an Inclusion of “Oral allergy syndrome”
		“Food allergy	Cited as an Inclusion of “Allergy or allergic reaction”
		“Allergy tests”	Cited as an Inclusion of “Diagnostic skin and sensitization tests”

**Fig. 4:**
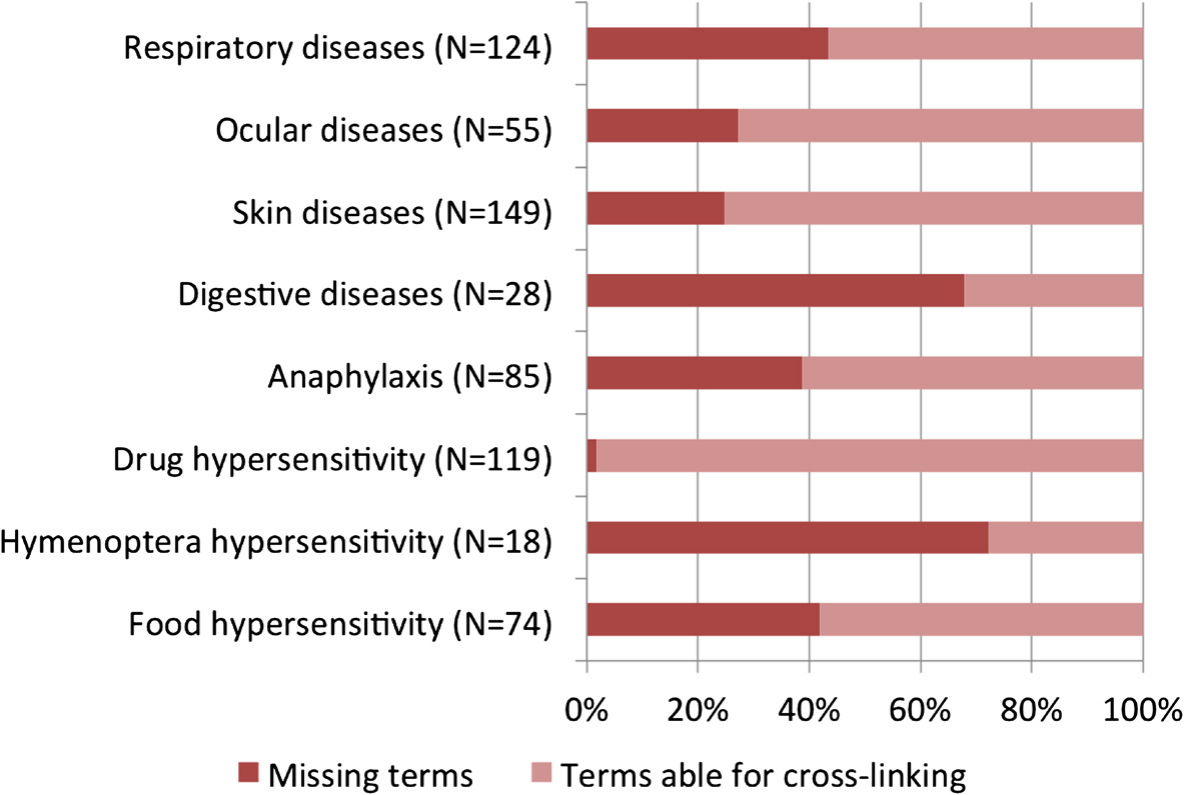
Proportion of missing terms for each hypersensitivity/allergic diseases group in the ICD-11 beta draft (October 2014 version)

The published Hypersensitivity/Allergic Diseases classification [8] counts with a total of 652 terms distributed in 5 main groups: Hypersensitivity/Allergic disorders involving the eye and the respiratory tract (179 terms), Hypersensitivity/Allergic disorders involving the skin and mucous membranes (149 terms), Hypersensitivity/Allergic disorders involving the digestive tract (28 terms), Anaphylaxis (85 terms) and Complex hypersensitivity/allergic disorders (119 terms for drug hypersensitivity, 18 terms Hymenoptera hypersensitivity and 74 terms for food hypersensitivity). Table 2 shows the results of the cross-linking terms process, in which 169 terms fit directly those classifications/codes listed in the ICD-11 beta draft (October 2014 version), and 26 were considered as “non-specific term”. Twenty-one terms could be linked to the Foundation by accessing the link Index, 7 were recorded as inclusions and 2 were cited just in the definition of the condition. The post-coordination was possible for 97 terms, mainly for drug hypersensitivity conditions. The proportion of the missing terms for hypersensitivity/allergic diseases main groups was the highest for Hymenoptera hypersensitivity and digestive diseases (Fig. 4).

**Table 2:** Cross-linking terms process: numbers of terms per main group of hypersensitivity/allergic diseases by pre and post-coordination, considered unspecific or did not fit properly to the ICD-11 beta draft (October 2014 version)

Main groups of Hypersensitivity/Allergic Diseases (number of terms listed in 652)		Pre-coordination			Post-coordination	No code fit properly	Non-specific term
		Linearization	Index to the Foundation	Cited as Inclusion			
		N (%)	N (%)	N (%)	N (%)	N (%)	N (%)
Respiratory diseases (124 terms)		36 (29)	0 (0)	3 (2)	15 (12)	11 (9)	12 (10)
Ocular diseases (55 terms)		20 (36)	2 (4)	0 (0)	6 (11)	5 (9)	5 (9)
Skin diseases (149 terms)		63 (42)	17 (11)	4 (3)	9 (6)	0 (0)	0 (0)
Digestive diseases (28 terms)		1 (4)	0 (0)	0 (0)	0 (0)	8 (29)	0 (0)
Anaphylaxis (85 terms)		5 (6)	0 (0)	0 (0)	14 (16)	4 (5)	7 (8)
Complex hypersensitivity/ allergic diseases	Drug hypersensitivity (119 terms)	26 (22)	2 (2)	0 (0)	53 (45)	7 (6)	0 (0)
	Hymenoptera hypersensitivity (18 terms)	3 (17)	0 (0)	0 (0)	0 (0)	2 (11)	0 (0)
	Food hypersensitivity (74 terms)	15 (20)	0 (0)	0 (0)	0 (0)	9 (12)	2 (3)

### Challenges of the cross-linking terms process

Improvements have been observed in the classification of some conditions in ICD-11 beta draft (October 2014 version) when compared to the ICD-10; however, the terms “hypersensitivity” and “allergy” are still misclassified in the ongoing revision, meaning that these concepts used routinely by the allergist community are still not globally accepted and may even be confusing. Both terms are in use mainly to describe specific conditions. It is notable that “hypersensitivity” is cited as an inclusion of “Adverse effects, not elsewhere classified (NOS)” meaning that it can be considered synonymous. It is also observed that the “hypersensitivity reactions” are considered disorders exclusively related to the lymphocytes number (Table 1). On the other hand, “Allergy or allergic reaction” is classified both as an inclusion, synonymous of “Hypersensitivity NOS”, “Idiosyncrasy NOS”, “Anaphylaxis NOS”, “Allergic shock NOS”, and under the same subchapter. On top of that, “Allergy or allergic reaction” excludes “Urticaria, angioedema and other urticarial disorders” from its panel although urticaria and angioedema can be IgE-mediated (allergen-induced) and are possible manifestations of anaphylaxis.

The cross-linking terms process demonstrated that some specific groups of hypersensitivity/allergic disorders, such as skin, ocular and drug hypersensitivities, fit better the current ICD-11 framework than other groups (*e.g.,* anaphylaxis, food and hymenoptera hypersensitivities) (Table 2), which probably reflects the specific work of the related TAGs. Regarding drug hypersensitivity, most of the conditions could be classified by post-coordination terms (*e.g.,* “Anaphylaxis due to systemic antibiotics” was classified by FN32.3 Drug-induced anaphylaxis + PC70 Systemic antibiotics or “Angioedema due to angiotensin converting enzyme (ACE) inhibitor” classified by FA75 Angioedema + PC7C.5 Angiotensin-converting-enzyme inhibitors).

The proportion of terms classified as “no code fit properly” was higher for hypersensitivity/allergic disorders of the digestive tract, which included exclusively digestive manifestations of food hypersensitivity. As an example, the result of searching “food-induced eosinophilic esophagitis” was “KA04.1 Neonatal eosinophilic oesophagitis”.

For respiratory hypersensitivity/allergic disorders, we observed a higher number of terms considered as “nonspecific”, with excessive generic extension and interpretation or able to cover many different conditions. The code/classification “DA05.Y Other specified vasomotor and allergic rhinitis” covered both Allergic and Non-allergic rhinitis such as Local Allergic Rhinitis, all Occupational Rhinitis (both caused and exacerbated by work), Rhinitis of the elderly, Gustatory Rhinitis, Hormonal-induced rhinitis and drug-induced rhinitis.

### Unmet needs of the cross-linking terms process for hypersensitivity/allergic diseases

The proportion of missing terms was notable for food, respiratory and hymenoptera venom hypersensitivity, mainly for the absence of disorder descriptions and lack of allergen references in the ICD-11 framework.

The considerable number of allergen references missing precludes even the post-coordination process. Most of important allergens diagnosed in the daily clinical practice of the allergist, such as (house) dust mite or milk, are absent or misclassified (Table 3). In the respiratory hypersensitivity/allergic disorders field, the only condition able to be properly cross-linked by pre-coordination mentioning an allergen is “DA05.2 Allergic rhinitis due to pollen”. For food allergens, the misclassification is remarkable. When we searched for the term “milk”, the conditions listed are basically related to mechanical injuries (*e.g.,* aspiration) and when the search is refined to “milk allergy”, no result is found. More than underlying the need of the inclusion of these terms in the new ICD edition, the current paper calls attention for the importance of involving the Allergy specialty in the revision process.

**Table 3:** Examples of allergens search in the ICD-11 beta phase (October 2014 version)

Terms searched	Results of searching process in the ICD-11 beta phase (October 2014 version)
“Mite”	“Infestation by mites”
	“Infestation of the skin by other specified parasitic mites”
	“Scrub (mite-borne) typhus”
“Milk”	“Intestinal obstruction due to inspissated milk”
	“Fetus and newborn affected or suspected to be affected by noxious influence transmitted via placenta or breast milk”
	“Other specified fetus and newborn affected or suspected to be affected by noxious influences transmitted via placenta or breast milk”
	“Fetus and newborn affected or suspected to be affected by noxious influence transmitted via placenta or breast milk, unspecified”
	“Neonatal aspiration of milk and regurgitated food”
	“Neonatal aspiration of milk and regurgitated food with respiratory symptoms”
	“Other specified neonatal aspiration of milk and regurgitated food”
	“Neonatal aspiration of milk and regurgitated food, unspecified”
	“Neonatal hyperbilirubinemia due to breast milk inhibitor of bilirubin conjunction”
	“Aspiration pneumonia (due to): milk”
	“Insufficient milk supply”
	“Delayed milk supply”
	“Oversupply of milk”
“Insect venom”	“Assault: Contact with person, animal or plant: Bitten, scratched or stung, non-venomous: Insect or bird”
	“Intentional Self Harm: Contact with person, animal or plant: Bitten, scratched or stung, non-venomous: Insect or bird”
	“Undetermined intent: Contact with person, animal or plant: Bitten, scratched or stung, non-venomous: Insect or bird”
	“Intent pending: Contact with person, animal or plant: Bitten, scratched or stung, non-venomous: Insect or bird”

For anaphylaxis, 40 % of terms were missing and the search of the term “anaphylaxis” in the ICD-11 beta draft showed 4 descriptions, mainly related to drug allergy: “drug-induced urticarial, angioedema and anaphylaxis”, “drug-induced anaphylaxis”, “anaphylaxis NOS” and “anaphylaxis due to medication in proper dose”. There are no references related with the severity grade of this condition or other associated allergies, which differs from the published Hypersensitivity/Allergic Diseases classification [7]. Furthermore, as highlighted before [4], anaphylaxis still resides under the same nonspecific “Injury, poisoning and certain other consequences of external causes” chapter, although some related conditions are described in other chapters such as “anaphylaxis due to contrast media” listed into the “Miscellaneous of urticarial disorders” of the Skin Disorders chapter.

All the conditions classified as “non specific term” and “no code fit properly” hamper the possibility of a better classification of hypersensitivity/allergic disorders, driving to the misclassification already highlighted in the ICD-10 structure. It suggests the need for updating and including more specific terms able to cover these conditions.

### Critical view of the cross-linking terms process for hypersensitivity/allergic diseases

Mapping is a labor-intensive process and needs to be updated regularly once the ICD-11 beta draft structure is not definite. One recognized limitation of this process is the possibility of broad interpretation for some terms, which can hamper the consistency of the classification system. In this way, the terms considered as “non specific” and “no code fit properly” may have to be tuned to better fit the purpose of a better classification for Hypersensitivity/Allergic diseases.

Since the online ICD-11 beta draft platform is not final and is updated regularly, the current results may not be reproduced if taken in a different time of analysis with the same methodology. Although this manuscript presents technical aspects of nomenclature and classification, we intended to introduce and simplify the complex concepts with the aim of becoming more familiar to the allergy community ICD end-users.

The results presented underline evidences of the need for changes in the ICD-11 framework and provides a baseline to expected improvements to allow the recognition of the diseases covered by the Allergy specialty. Although we are aware of the enormous structural changes and efforts needed to this end, having Hypersensitivity/allergic diseases well classified in the ICD-11 may have important impact for the visibility of our specialty. It is important to recognize that in addition to the highlighted areas, appropriately coded health data are important for a number of clinical reasons. The most important clinical utility of coded information is to support appropriate computerized decision support around ordering investigations, medicines management and bundling of care, and related performance management.

**Fig. 5:**
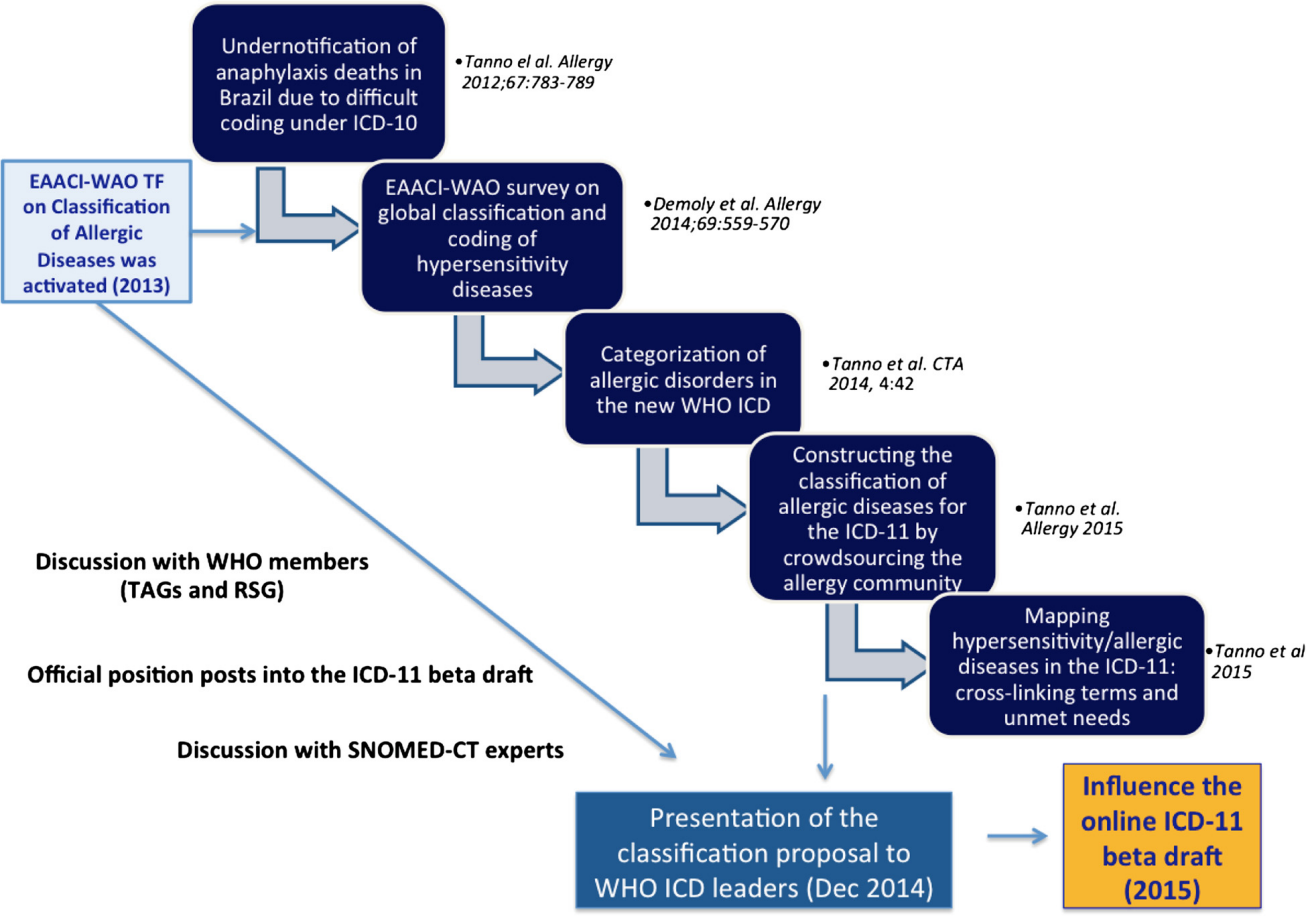
The Joint Allergy Academies strategies for a better classification of hypersensitivity/allergic diseases in the ICD-11

The current manuscript had the hypersensitivity/allergic diseases classification validated by crowdsourcing as the basis for its construction. Since the crowdsourcing process has been supported and acknowledged by a Joint Allergy Academies, composed by the EAACI, the World Allergy Organization (WAO) and the American Academy of Asthma Allergy and Clinical Immunology (AAAAI), we believe that the expected implementations in the ICD-11 by the allergy perspective (Fig. 5) will count with a harmonized point of view.

## Conclusions

### Outcomes of the cross-linking terms process for the hypersensitivity/allergic diseases

Many of the Hypersensitivity/Allergic diseases classification terms contain clinical information encoded in the ICD-11 beta draft and the cross-linking process was possible by pre-coordination to some conditions in detriment to specific ones in which the post-coordination fit better. However, we still notice an important degree of missing and “non specific” terms.

The unspecific results of searching “hypersensitivity” and “allergy” terms demonstrate that the Allergy specialty has still concepts not universally accepted. The Allergy specialty involvement in the current revision would contribute to upgrade the awareness of the hypersensitivity/allergic conditions in the ICD revision.

The proposed strategy of cross-linking terms and the results of this process can actively contribute to updating the hypersensitivity and allergic conditions classification in the ICD-11 beta revision by objectively demonstrating which entries can fit the ongoing ICD-11 structure, indicating the categories which should be improved to have a reliable understanding as well as bringing up the missing categories. The results of the process underline the need for either a new chapter in ICD-11 possibly entitled Hypersensitivity / Allergic Disorders or at the very least the aggregation of all such diseases under the “Diseases of Immune System” chapter in order for the overlaps to be double parented to the appropriate ‘system’ chapters.

## Abbreviations

AAAAI: American Academy of Allergy, Asthma and Immunology
ACE: angiotensin converting enzyme
DSM: Diagnostic and Statistical Manual of Mental Disorders
EAACI: European Academy of Allergy and Clinical Immunology
ICD: International Classification of Diseases
RSG: Revising Steering Group
SNOMED CT: Systematized Nomenclature of Medicine Clinical Terms
TAGs: Topic Advisory Groups
WAO: World Allergy Organization
WHO: World Health Organization
